# 14-3-3-Pred: improved methods to predict 14-3-3-binding phosphopeptides

**DOI:** 10.1093/bioinformatics/btv133

**Published:** 2015-03-03

**Authors:** Fábio Madeira, Michele Tinti, Gavuthami Murugesan, Emily Berrett, Margaret Stafford, Rachel Toth, Christian Cole, Carol MacKintosh, Geoffrey J. Barton

**Affiliations:** ^1^Division of Computational Biology,; ^2^Division of Cell and Developmental Biology,; ^3^MRC Protein Phosphorylation Unit,; ^4^Division of Signal Transduction Therapy,; ^5^Division of Biological Chemistry and Drug Discovery, College of Life Sciences, University of Dundee, Dundee DD1 5EH, Scotland, UK

## Abstract

**Motivation:** The 14-3-3 family of phosphoprotein-binding proteins regulates many cellular processes by docking onto pairs of phosphorylated Ser and Thr residues in a constellation of intracellular targets. Therefore, there is a pressing need to develop new prediction methods that use an updated set of 14-3-3-binding motifs for the identification of new 14-3-3 targets and to prioritize the downstream analysis of >2000 potential interactors identified in high-throughput experiments.

**Results:** Here, a comprehensive set of 14-3-3-binding targets from the literature was used to develop 14-3-3-binding phosphosite predictors. Position-specific scoring matrix, support vector machines (SVM) and artificial neural network (ANN) classification methods were trained to discriminate experimentally determined 14-3-3-binding motifs from non-binding phosphopeptides. ANN, position-specific scoring matrix and SVM methods showed best performance for a motif window spanning from −6 to +4 around the binding phosphosite, achieving Matthews correlation coefficient of up to 0.60. Blind prediction showed that all three methods outperform two popular 14-3-3-binding site predictors, Scansite and ELM. The new methods were used for prediction of 14-3-3-binding phosphosites in the human proteome. Experimental analysis of high-scoring predictions in the FAM122A and FAM122B proteins confirms the predictions and suggests the new 14-3-3-predictors will be generally useful.

**Availability and implementation:** A standalone prediction web server is available at http://www.compbio.dundee.ac.uk/1433pred. Human candidate 14-3-3-binding phosphosites were integrated in *ANIA: ANnotation and Integrated Analysis of the 14-3-3 interactome* database.

**Contact:** cmackintosh@dundee.ac.uk or gjbarton@dundee.ac.uk

**Supplementary information:** Supplementary data are available at *Bioinformatics* online.

## 1 Introduction

The 14-3-3 phosphoprotein-binding proteins interact with many intracellular targets. Changes in the engagement of 14-3-3s with different sets of target phosphoproteins cause coordinated shifts in cellular behaviour in response to growth factors and other stimuli (Bridges and Moorhead, 2005; Johnson *et al*., 2010, 2011; MacKintosh, 2004). 14-3-3s are boat-shaped dimers that dock onto specific pairs of phosphorylated Ser and Thr residues (Obsil and Obsilová, 2011). The phosphosite pairs are often located in tandem on the same target protein and are typically >15 amino acid residues apart to allow engagement with both docking sites in the central groove of the 14-3-3 dimer (Yaffe, 2002; Yaffe *et al.*, 1997; Zhu *et al.*, 2005). The 14-3-3s also have strong preferences with respect to the sequences immediately flanking the phosphorylated residues (Yaffe, 2002; Yaffe *et al.*, 1997; Zhu *et al.*, 2005).

Early analyses of known 14-3-3-binding sites indicated R(S)X1,2(pS)X(P) as a 14-3-3-binding motif (Muslin *et al.*, 1996; MacKintosh, 2004). Later screening of libraries for phosphopeptides that displayed optimal binding to 14-3-3s identified two consensus 14-3-3-binding motifs, namely Mode I (RSX(pS/T)XP) and Mode II (RX(F/Y)X(pS)XP), with subtle preferences and negative determinants for the X residues (Yaffe, 2002; Yaffe *et al.*, 1997; Obsilová *et al.*, 2008). These sequence motifs served as the basis for a position-specific scoring matrix (PSSM) to predict potential 14-3-3-binding phosphosites in Scansite (Obenauer, 2003). A more recent survey showed that most experimentally determined 14-3-3-binding sites (dubbed gold standards) conform to mode I motifs, having at least one basic residue in the positions −3 to −5, relative to the phosphoSer/Thr and never a +1 proline. However, the proline at +2 is found in fewer than 50% of cases and often the serine in the −2 position relative to the 14-3-3-binding phosphosite is a residue that is annotated as phosphorylated (Johnson *et al.*, 2010). Additionally, Mode III sites, in which the phosphorylated residue is the penultimate residue in the C-terminal tail of a protein target, have also been reported (Coblitz *et al.*, 2006; Panni *et al.*, 2011).

Recently, a further striking pattern was identified in the human 14-3-3 interactome. The majority of well-defined human 14-3-3-binding phosphoproteins were discovered to be 2R-ohnologues (Tinti *et al.*, 2012). This means that they belong to protein families of two to four members that were generated by the two rounds of whole-genome duplication (2R-WGD), which marked the evolutionary origins of the vertebrate animals ∼500 million years ago (Huminiecki and Heldin, 2010; Makino and McLysaght, 2010). Most of the new genes were negatively selected and lost. However, those that were retained in families of two to four members are highly enriched in signalling proteins that bind to 14-3-3s (Huminiecki and Heldin, 2010; Tinti *et al*., 2012). In case studies, protein families were identified whose members share one 14-3-3 binding site in common (termed the ‘lynchpin’). Lynchpins also align with a serine or threonine residue in the pro-orthologue proteins from the pre-2R-WGD invertebrate chordates, *Branchiostoma* (amphioxus, lancelet) and *Ciona* (tunicates, sea squirts). In contrast, the second sites may differ on different family members and may be phosphorylated by different protein kinases. These findings led to the proposal that 14-3-3 dimers may have played a mechanistic role in the regulatory divergence of 2R-ohnologue families: The lynchpin hypothesis proposes that conservation of one ‘lynchpin’ 14-3-3-binding site gave the freedom for the second site to change and perhaps become a consensus site for phosphorylation by a different protein kinase (Johnson *et al.*, 2011). The resulting protein families therefore operate as ‘signal multiplexing’ systems that are regulated by a wider array of protein kinases than would be possible if the function were performed by only a single protein.

Currently, the Scansite 14-3-3 predictor (Obenauer, 2003) is the most commonly used software tool to identify potential 14-3-3-binding phosphosites. Scansite was trained on peptide libraries derived from a limited number of experimentally defined 14-3-3-binding sites, but these training datasets no longer accommodate the diversity of known 14-3-3-binding phosphopeptides. Another source of information on 14-3-3-binding sites is the ‘eukaryotic linear motif’ database ELM (Puntervoll, 2003). ELM uses regular expressions and context-based filtering to derive pattern probabilities based on a few dozen Mode I, Mode II and non-consensus motifs.

There are now >2000 phosphoproteins that have been found to display affinity for 14-3-3 in high-throughput proteomics experiments (Jin *et al*., 2004; Nishioka *et al*., 2012; Pozuelo Rubio *et al*., 2004). Accordingly, there is a need to extend predictors to include 14-3-3 binding sites that do not conform to Mode I binding and to test the signal multiplexing hypothesis. A more comprehensive picture of potential 14-3-3 binding sites would help to define how the complete 14-3-3-interactome system works. The ANIA (ANnotation and Integrated Analysis of the 14-3-3 interactome) web service and database (Tinti *et al.*, 2014) integrates multiple datasets on 14-3-3-binding phosphoproteins and provides an up-to-date gold-standard dataset of experimentally determined 14-3-3-binding phosphosites of all known Modes. In this article, three new classifiers of 14-3-3-binding sites are described that have been trained on the ANIA gold-standard dataset. The new predictors are compared with Scansite and ELM, predictions for the human phosphoproteome performed and a couple of high-scoring sites experimentally tested.

## 2 Methods

### 2.1 Data collection and preprocessing

The human proteome was retrieved from the UniProt database (June 2013 release) and all Ser/Thr residues located in every protein sequence. A collection of annotated phosphoSer/Thr sites (phosphoproteome) was gathered from PhosphoSitePlus (October 2013 release) (Hornbeck *et al.*, 2004).

A list of 300 experimentally determined 14-3-3-binding phosphosites was collected from ANIA (Tinti *et al.*, 2014) and further extended from the literature to give 322 gold-standard 14-3-3-binding sites (*POS*) (Supplementary Table S1). A negative dataset (*NEG*) (Supplementary Table S2) was assembled from the literature cited in Johnson *et al*. (2010), resulting in 93 phosphosites. To prepare balanced sets of *POS* and *NEG* examples, 230 additional likely non-binding sites were randomly selected from a subset of proteins for which two 14-3-3-binding sites had been experimentally defined. Although the likely *NEG* sites are located in 14-3-3-binding proteins, these sites are thought unlikely to bind 14-3-3s since there is currently no evidence of proteins that bind 14-3-3 through multiple pairs of phosphosites. The resulting *POS* and *NEG* datasets comprised balanced numbers of phosphopeptides that were further processed for training of the classifiers.

To explore motif patterns that are in agreement with the modes of binding previously proposed, five non-symmetrical motif windows around the phosphoSer/Thr site were defined, including [−3:1], [−4:2], [−5:3], [−6:4] and [−7:5]. These motif windows ranged from 4 to 12 residues in width not including the central phosphoSer/Thr residue. The peptides in the *POS* and *NEG* datasets were also filtered for sequence redundancy at a range of identity thresholds for all pairwise peptide comparisons. When working with small peptides, a single amino acid difference can be critical for determining specificity. Thus, determining redundancy in short peptides is not straightforward. In this article, redundancy is defined by differences of 1…*k/*2 amino acids, where *k* is length of the peptide. Thus, redundancy thresholds ranged from a minimum of one residue difference up to half of the size of the motif window (equivalent to 50% redundancy level). For example, for motif window [−6:4] that comprises 10 residues, five levels of redundancy were investigated with a minimum number of differences ranging from one to five. Since the number of redundancy thresholds investigated depends on the size of the motif window in analysis, all combinations of windows and redundancy thresholds were tested in model training and testing.

To reduce the risk of bias, the resulting pairs of balanced *POS* and *NEG* datasets from different combinations of motif windows and redundancy thresholds were further split into two independent training and testing subsets. This gave 240 (75%) and 78 (25%) peptides for training and testing, respectively. After selecting the best overall models in training and testing, final methods were trained using the full non-redundant training and testing subsets, comprising 318 *POS* and 318 *NEG* peptide examples in total (100%) (Supplementary Tables S1 and S2). An additional independent and ‘blind’ test dataset (*BLIND*) comprising 38 experimentally defined 14-3-3-binding sites was collected from the literature (Supplementary Table S3). Following the same strategy used for preparing the training datasets, 32 likely non-binding phosphosites were selected as *BLIND* negatives (Supplementary Table S4).

### 2.2 Classification methods

#### 2.2.1 Artificial neural network

Artificial neural network (ANN) models were trained using the R package RSNNS (Bergmeir and Benítez, 2012) and the Stuttgart Neural Network Simulator (SNNS; http://www.ra.cs.uni-tuebingen.de/SNNS). For ANN training, each of the 20 different amino acids was encoded as a binary vector of length 20. For example, Ala was encoded as [1,0,0,0,0,0,0,0,0,0,0,0,0,0,0,0,0,0,0,0], whereas Arg was encoded as [0,1,0,0,0,0,0,0,0,0,0,0,0,0,0,0,0,0,0,0]. This pattern was followed for all 20 amino acids, whereas gaps or other ambiguous amino acids were encoded as [0,0,0,0,0,0,0,0,0,0,0,0,0,0,0,0,0,0,0,0]. Accordingly, phosphopeptides of length *k* (4 ≤ *k* ≤ 12) were encoded by vectors of length 20*k*. The final ANN model had 20 input nodes, a single hidden layer with 20 nodes and one output layer with one output node. Training was performed by the backpropagation algorithm with momentum term (‘Backpropmomentum’), learning parameter *η* = 0.2 and momentum term *µ* = 0.05.

#### 2.2.2 Position-specific scoring matrix

PSSMs were implemented in Python (http://www.python.org) and assembled by adapting the procedure described by Ferrari *et al.* (2011). Amino acid frequency matrices were derived from *POS* and *NEG* datasets and from a background (*BGD*) dataset, made up of all peptides that have annotated phosphoSer/Thr sites (phosphoproteome). For each motif window of length *k* of the alignment (4 ≤ *k* ≤ 12), a PSSM was assembled with 21 rows [20 amino acids plus gaps or ambiguous (X), AA = (A, R, N, D, C, Q, E, G, H, I, L, K, M, F, P, S, T, W, Y, V, X)] and *k* columns, where the values represent the frequency of amino acid *i* {*i* ∈ AA} at the *j*th position {*j* = 1…*k*} in the multiple alignment of all peptides. Equation (1) defines the final score *S*(*p*) assigned to each queried phosphopeptide *p,* which is calculated by adding up the scores for all the positions, where

(1) $$S(p)=\sum_{j}^{k}\frac{POS_{i,j}-NEG_{i,j}}{BGD_{i,j}}$$

*POS_i,j_*, *NEG_i,j_* and *BGD_i,j_* are the frequency values for amino acid *i* at position *j*, in the *POS*, *NEG* and *BGD* matrices, respectively.

#### 2.2.3 Support vector machines

Support vector machine (SVM) models were trained and parameterized using the Python module PyML (http://pyml.sourceforge.net), which contains a set of non-linear kernels specifically developed for training and classification of biological sequences (Ben-Hur *et al.*, 2008). The final SVM model employed the weighed-degree kernel (Sonnenburg *et al.*, 2005), with soft margin constant *C*, which specifies the degree of separation between the two training classes of support vectors in the hyperplane was set to one. Lastly, the cosine kernel was applied to normalize the kernel values.

### 2.3 Feature selection for the ANN models

Two independent alphabet reduction systems were tried. Both methods grouped the 20 amino acids in 10 classes according their physicochemical properties and were encoded as an orthogonal 10-length binary (Li *et al.*, 2003; Livingstone and Barton, 1993).

Further features were also explored as inputs to the ANN model. Protein secondary structure predictions and solvent accessibility were computed by Jpred (Cole *et al.*, 2008), which provides predictions of α-helix, β-strand, random coil and solvent accessible or buried. In addition to the 20-length binary vector of amino acid encodings, every residue position including the central Ser/Thr was encoded as a 5-length binary vector or alternatively encoded as a vector of raw Jpred prediction scores [0.0:1.0], resulting in a vector of length 20*k* + 5(*k* + 1). Similarly, three methods for predicting natively unstructured/disordered regions in proteins (Dosztányi *et al.*, 2005; Linding, 2003; Linding *et al.*, 2003) were computed using the JABAWS package (Troshin *et al.*, 2011). Peptide motifs were classified as disordered or structured by four methods and were encoded as a binary vector of length 2, which resulted in an encoding vector of length 20*k* + 2(4). IUPred prediction scores ≥0.5 were used to define disordered regions, whereas for GlobPlot, the Dydx algorithm with a threshold of ≥0.0 was used. Regions predicted by both DisEMBL algorithms: HOTLOOPS and REM465 were considered for disorder classification.

### 2.4 Evaluation methods

The performance of each classifier was evaluated by Jackknife (leave-one-out cross-validation) on the training and testing data, before a final test on the *BLIND* dataset. The performance of each method was assessed by receiver operating characteristic (ROC) curves, which were plotted at various thresholds (Fawcett, 2004). The area under the ROC curve (AUC) (Sonego *et al.*, 2008) was used as the primary performance measure. Additional standard metrics were calculated for each method including sensitivity (SN, equivalent to recall) (Equation 1), specificity (SP) (Equation 2), positive predictive value (PPV, equivalent to precision) (Equation 3), accuracy (ACC) (Equation 4) and Matthews correlation coefficient (MCC) (Equation 5), where TP, FP, TN, FN denote the number of true positives, false positives, true negatives and false negatives, respectively.

(1) $$\text{SN}=\frac{\text{TP}}{\text{TP}+\text{FN}}$$

(2) $$\text{SP}=\frac{\text{TN}}{\text{TN}+\text{FP}}$$

(3) $$\text{PPV}=\frac{\text{TP}}{\text{TP}+\text{FP}}$$

(4) $$\text{ACC}=\frac{\text{TP}+\text{TN}}{\left(\text{TP}+\text{TN}+\text{FP}+\text{FN}\right)}$$

(5) $$\text{MCC}=\frac{\text{TP}\times \text{TN}-\text{FP}\times \text{FN}}{\sqrt{\left(\text{TP}+\text{FP})(\text{TP}+\text{FN})(\text{TN}+\text{FP})(\text{TN}+\text{FN}\right)}}$$

Evaluation and statistical analysis was performed in the R statistical language (http://www.r-project.org) and ROCR package (Sing *et al.*, 2005). Sample correlation analysis was performed by the Pearson correlation coefficient (*r*). For two-sample paired tests, the Wilcoxon–Mann–Whitney test and the Student’s *t*-test were performed. The null hypothesis was inferred at a 95% level of confidence.

### 2.5 Biochemical methods

The cDNA encoding human FAM122A (Q96E09) was amplified from IMAGE consortium EST clone 6182641 (coding for NM_138333.3 CDS) and FAM122B (Q7Z309) was from IMAGE consortium EST clone 3841054 (coding for NM_145284.3 CDS). Three isoforms of FAM122C (Q6P4D5) were cloned: NP_620174.1 amplified from IMAGE clone 4699951 (152 residues); Q6P4D5.1, amplified from IMAGE clone 5229041 (195 residues) and AAH65225.1, amplified from IMAGE clone 5724414 (96 residues). cDNAs were cloned as BamHI/NotI inserts into the multiple cloning site of pcDNA5 FRT/TO that adds a C-terminal GFP tag to the expressed protein. Mutants were made using polymerase chain reaction mutagenesis and DNA sequencing was performed by The Sequencing Service, University of Dundee (www.dnaseq.co.uk). Plasmids are available from the MRC-PPU reagents website (mrcppureagents.dundee.ac.uk).

Proteins were isolated using GFP-Trap® (ChromoTek) from lysates of transfected human embryonic kidney 293 (HEK293) cells, using a lysis buffer that preserves their *in vivo* phosphorylation status. The isolated proteins were tested for retention of co-purified endogenous 14-3-3 proteins (K19 pan-14-3-3 antibody, Santa Cruz Biotech) and for their ability to bind directly to 14-3-3s in Far-Western overlays, as described in Tinti *et al.* (2014). Where indicated, isolated proteins were dephosphorylated, or not, as described in Tinti *et al.* (2014) prior to analysis of their interaction with 14-3-3.

## 3 Results and discussion

### 3.1 Development and evaluation of 14-3-3 classifiers

Three new 14-3-3 classifiers were developed in this work. Data preprocessing was performed, so that all combinations of motif window length and redundancy thresholds were evaluated in model training and model testing. The comparison of the AUC scores by Jackknife for the resulting classifiers showed that the highest performance was achieved at a redundancy threshold of at least one residue difference (sequence identity <90%), for a motif region spanning from −6 to 4. A motif window [−6:4] agrees with observed 14-3-3-binding modes (Johnson *et al.*, 2010) and performed better in this study than [−7:7], which has been previously selected for this kind of classification task (Miller *et al*., 2008; Obenauer, 2003).

**Fig. 1.**
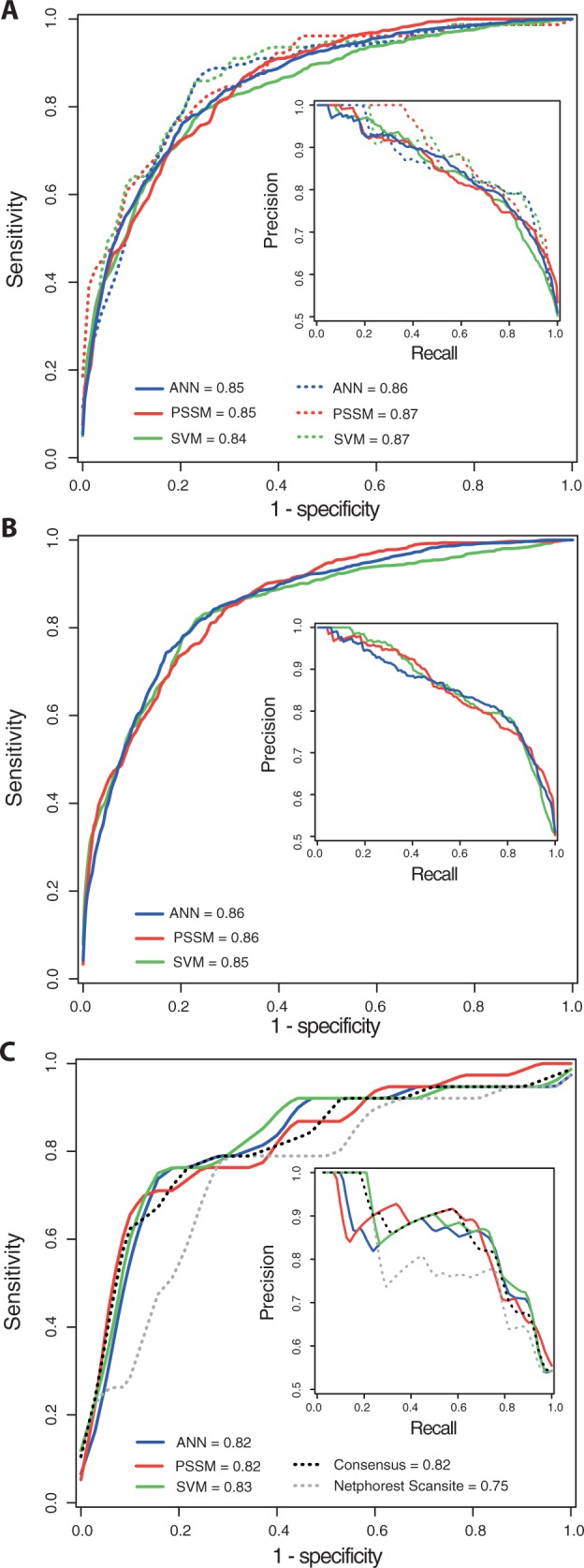
Performance of the classifiers tested by Jackknife. ROC curves and the AUC scores for (**A**) model training (bold line) and model testing (dotted line); (**B)** final models and (**C**) comparison to Netphorest Scansite on the *BLIND* dataset. ANN, PSSM and SVM models were trained at the redundancy level of at least one residue difference and for a [−6:4] motif window

As shown in Figure 1A, all three methods performed similarly well in model training and model testing, with AUC scores ranging from 0.84 (for the SVM in model training) to 0.87 (for PSSM and SVM in model testing). Figure 1B shows the performance of the final models trained using balanced and non-redundant *POS* and *NEG* datasets (318 *POS* and 318 *NEG* peptide examples), generated by combining non-redundant training and testing sub-sets. ANN and PSSM showed an AUC of 0.86, whereas the SVM showed an AUC of 0.85. Although globally the performance of the final methods is not significantly different, the ANN presented the highest MCC score of 0.59 ± 0.01 (SD), accuracy (ACC) of 79.6 ± 0.6% and a PPV of 79.8 ± 1.6%.

Since phosphorylated Ser/Thr usually lie at the protein surfaces enabling kinase/phosphatase activity, as well as regulation by the 14-3-3s (Vandermarliere and Martens, 2013), additional features such as secondary structure, solvent accessibility, and protein disorder were tested to see their effect on performance, as was feature-selection by amino acid alphabet reduction. Although these approaches have proved useful in a number of classification tasks (e.g. Aytuna *et al*., 2005; McDowall *et al*., 2009), here they added complexity but did not give a significant improvement in the performance of the methods developed here (data not shown).

### 3.2 Comparison with other predictors

The performance of the new classification methods developed here was compared with Scansite (Obenauer, 2003) and ELM (Puntervoll, 2003), using an additional dataset (the *BLIND* set) that was not used for training the methods. The *BLIND* dataset comprises 38 experimentally defined 14-3-3-binding phosphopeptides and 32 non-14-3-3-binding sites. Raw Scansite prediction scores were obtained by querying Netphorest (Miller *et al.*, 2008) that fully implements the original Scansite PSSM. Categorical classification scores were also obtained for each *BLIND* phosphoprotein, from the Scansite2 (Obenauer, 2003), Scansite3 (unpublished work by T.Ehrenberger, 2012) and ELM web servers (Dinkel *et al.*, 2013). Scansite2 provides prediction scores based on 14-3-3 Mode I motifs that fall into three stringency levels: *high*, *medium* and *low*. Scansite3, a Java implementation of Scansite2, enables search for a fourth stringency level: *minimum*. ELM uses context-based filtering and text mining to improve the accuracy of assigned pattern-matching probabilities based on Mode I and Mode II 14-3-3-binding as well as non-consensus 14-3-3-binding. Overall, any phosphoSer/Thr site for which a prediction score was provided (at a particular stringency level, in the case of Scansite) was considered to be classified as 14-3-3-binding. All the remaining sites were classified as non-14-3-3-binding. Although other methods exist to predict 14-3-3 binding sites (Chan *et al.*, 2011; Panni *et al.*, 2011), no software or pre-computed predictions were available for comparison to the methods developed here.

As listed in Table 1, all three methods developed here showed higher MCC scores (up to 0.60 for ANN and PSSM), when compared Netphorest Scansite, Scansite2 and Scansite3 at different stringency levels and ELM (up to 0.52 for Scansite2 *low* and ELM). Indeed, the new methods present the best accuracy (ACC of 80.0% for the ANN and 78.6% for PSSM and SVM), compared with accuracies of the other predictors, which are generally lower than 75.7% (obtained for Scansite2 *low*). The performance scores of the new methods agree with the performance observed in model training and testing (Fig. 1). However, the PPV observed for the *BLIND* dataset is higher (from 79.8% for the final models compared with ≥85.3% PPV for the *BLIND* dataset), which might be an outcome of the unbalanced composition of the *BLIND* dataset. A consensus predictor achieved 0.61 MCC and 80.0% ACC by averaging the scores from the three methods. Both Scansite2 and Scansite3 *high* stringency showed 100% PPV and 100% specificity (SP). This results from the fact that both methods incorrectly classify most sites as non-14-3-3 binding and give small numbers of TP and FP (zero FP in this particular case) but high FN. In terms of sensitivity (SN), the best method was Scansite3 *minimum* with 84.2% SN, compared with the ANN that showed 76.3% SN. Here, Scansite3 *minimum* correctly predicted 32 out of 38 *POS* examples, whereas the ANN correctly predicted 29/38. Although Scansite predictors show higher SN and SP, the new predictors show a better balance between these two metrics, which leads to superior ACC and MCC scores.

**Table 1.** Comparison of the predictors developed in this study with Scansite and ELM, for an external *BLIND* dataset comprising 38 literature-curated 14-3-3-binding sites and 32 non-binding sites

Predictor	TP	FP	TN	FN	SN (%)	SP (%)	PPV (%)	ACC (%)	MCC *
Consensus^a^^,^^b^	28	4	28	10	73.7	87.5	87.5	**80.0**	**0.61**
ANN^a^	29	5	27	9	76.3	84.4	85.3	80.0	0.60
PSSM^a^	26	3	29	12	68.4	90.6	89.7	78.6	0.60
SVM^a^	27	4	28	11	71.1	87.5	87.1	78.6	0.59
Netphorest Scansite^c^	28	7	25	10	73.7	78.1	80.0	75.7	0.52
Scansite2 *low*^d^	28	7	25	10	73.7	78.1	80.0	75.7	0.52
ELM^d^	24	4	28	14	63.2	87.5	85.7	74.3	0.52
Scansite3 *low*^d^	27	7	25	11	71.1	78.1	79.4	74.3	0.49
Scansite3 *minimum*^d^	32	13	19	6	84.2	59.4	71.1	72.9	0.45
Scansite2 *high*^d^	12	0	32	26	31.6	100.0	100.0	62.9	0.42
Scansite2 *medium*^d^	17	4	28	21	44.7	87.5	81.0	64.3	0.35
Scansite3 *high*^d^	9	0	32	29	23.7	100.0	100.0	58.6	0.35
Scansite3 *medium*^d^	17	4	28	21	44.7	87.5	81.0	64.3	0.35

SN, sensitivity; SP, specificity; NPV, negative predictive value; ACC, accuracy. The table is sorted by MCC score.

^a^The results shown were calculated based on optimal thresholds derived from accuracy/cut-off plots for the final models. The cut-offs are 0.55, 0.80, 0.25 and 0.50, for ANN, PSSM, SVN and Consensus, respectively.

^b^The consensus predictor averages the scores obtained by the three methods: ANN, PSSM and SVM.

^c^Scansite PSSM prediction scores were obtained by querying Netphorest. An optimal cut-off of 0.15 resulted in the balanced performance observed for Scansite2 *low.*

^d^Based on categorical classification of the queried phosphoproteins.

*The significance level of each method’s MCC score was assessed against the MCC score of the consensus predictor by computing a distribution of MCC scores for 100 bootstrap replicates with replacement, randomly selecting examples from the *BLIND* dataset. Underlined MCC scores indicate that the method is significantly worse than the consensus predictor (*P* < 0.05), whereas double underline indicates high significance (*P* < 0.001).

Two-sample sequence analysis of the final *POS* and *NEG* datasets revealed that Mode I is indeed the most common, accounting for ∼46% enrichment of Arg at position −3 and ∼31% enrichment of Pro at +2 position. Additionally, poorer enrichment of Ser and Leu at positions −2 and +1, respectively, as well as depletion of Pro at +1, is also observed. A similar profile is observed for the *BLIND* dataset, which might explain why Scansite *low* and *minimum* correctly classifies *POS* examples equally well as the consensus predictor. However, non-consensus binding motifs are better covered by the methods introduced here since the consensus motifs I and II represent less than 30% of *POS* in both training and blind datasets. The correlation between the consensus predictor and Netphorest Scansite scores, for prediction of 14-3-3-binding phosphopeptides in the human proteome (Section 3.3), is only *r* = 0.65 (*P* < 0.001), indicating that a fair number of peptides produce discordant predictions by Scansite and the methods developed here.

Overall, based on the performance measures and benchmark results listed in Table 1 and Figure 1C, all methods introduced in this study outperform the previous predictors with Scansite2 *low* and ELM the closest rivals. The consensus predictor is significantly better than all the Scansite predictors and ELM, based on the MCC scores obtained for the *BLIND* dataset (*P* < 0.05). As the exact peptide datasets used for training Scansite are not known, it is likely that the real performance of Scansite will be lower, as some of the tested examples could have been used for its training. Similarly, ELM adds literature annotation for known 14-3-3-binding phosphosites when available, so its prediction performance is perhaps over optimistic. In fact, ∼60% (15/24) TP were annotated from the literature, making this benchmark evaluation moderately biased in favour of ELM. Intriguingly, Scansite2 and Scansite3 presented some classification differences at *high* and *low* stringency levels. Whether this difference is the result of the new implementation of Scansite3, potentially setting new underlying stringency thresholds, or due to the addition of a *minimum* stringency level is not clear.

### 3.3 Prediction and experimental testing of 14-3-3-binding phosphosites in the human proteome

All 1 543 965 Ser/Thr residues in the 20 245 proteins of the human proteome as released in June 2013 were considered as potential 14-3-3 sites and ranked according to the methods developed here. The consensus classifier predicted a total of 75 891 potential binding sites in 17 214 proteins. This corresponds to 4.9% of all Ser/Thr sites with an average of four sites per protein. Considering only the set of 117 640 proteins for which phosphoSer/Thr sites have already been annotated, the number of predicted sites falls to 10 881 in 5483 proteins. This corresponds to 9.2% of all Ser/Thr sites in the phosphoproteome and an average of two candidate phosphosites per protein, which reduces the potential number of FP, since proteins known to be phosphorylated potentially bind to 14-3-3 dimers. This approach makes it more amenable for prioritizing experimental investigation. As shown below, two high-ranking ANN predictions were further tested by experiment.

Table 2 lists the top 20 high-scoring candidates’ sites predicted by the three methods on the phosphoproteome. Predicted proteins include sperm-specific antigen 2 (3rd); sorbin and SH3 domain-containing protein 1 (5th); negative elongation factor E (6th); E3 ubiquitin-protein ligase HUWE1 (17th); E3 ubiquitin-protein ligase UBR4 (18th) and Centrosomal protein of 170 kDa (19th): all of which had been previously detected in 14-3-3-binding capture experiments (Tinti *et al.*, 2014; Wang *et al.*, 2011) but whose 14-3-3-binding sites remained elusive. High-scoring predictions by the consensus predictor support that these proteins partner with 14-3-3s; however, further experiments have to be performed to validate these candidate binding sites.

**Table 2.** Top 20 high scoring predictions and their respective scores

Rank	Protein	Description	Site	Motif	Consensus^a^
1	PPP1R3G	Protein phosphatase 1 regulatory subunit 3G	86	CRARSF**S**LPAD	1.97
2	**FAM122A**^b^^,^^c^	**Family with sequence similarity 122A**	37	GLRRSN**S**APLI	1.88
3	SSFA2^b^	Sperm-specific antigen 2	739	PLRRSQ**S**LPTT	1.87
4	ALOX12^c^	Arachidonate 12-lipoxygenase, 12S-type	246	LLRRST**S**LPSR	1.83
5	SORBS2^b^	Sorbin and SH3 domain-containing protein 2	259	FRKRRK**S**EPAV	1.77
6	NELFE^b^	Negative elongation factor E	251	PFRRSD**S**FPER	1.75
7	ANKRD63	Ankyrin repeat domain-containing protein 63	332	GLRRRS**T**APDI	1.74
8	SECISBP2L^c^	Selenocysteine insertion sequence-binding protein 2-like	251	GRRRRA**S**HPTA	1.72
9	FAM13A^c^	Family with sequence similarity 13A	741	MRQRSN**T**LPKS	1.72
10	FAM189A2^c^	Family with sequence similarity 189A2	275	LRTRSK**S**DPVL	1.71
11	**FAM122B**^b^^,^^c^	**Family with sequence similarity 122B**	25	TLRRSS**S**APLI	1.67
12	TRAK2^c^	Trafficking kinesin-binding protein 2	420	TRGRSI**S**FPAL	1.67
13	CEP57^c^	Centrosomal protein of 57 kDa	55	DLRRSP**S**KPTL	1.66
14	GOLGA5	Golgin subfamily A member 5	116	FVRRKK**S**EPDD	1.66
15	CISD2^c^	CDGSH iron-sulphur domain-containing protein 2	106	RCWRSK**T**FPAC	1.66
16	TBC1D22A^c^	TBC1 domain family member 22A	167	PLQRSQ**S**LPHS	1.65
17	HUWE1^b^	E3 ubiquitin-protein ligase HUWE1	649	MRRRRS**S**DPLG	1.65
18	UBR4^b^	E3 ubiquitin-protein ligase UBR4	2715	NKRRHV**T**LPSS	1.62
19	CEP170^b^	Centrosomal protein of 170 kDa	644	GERRRR**T**LPQL	1.60
20	TMEM40	Transmembrane protein 40	137	GLRRRG**S**DPAS	1.59

Proteins that have shown affinity to 14-3-3 in high-throughput experiments and 2R-ohnologue members were identified by querying ANIA. FAM122A and FAM122B were experimentally verified to bind 14-3-3 in this study.

^a^The consensus predictor averages the scores obtained by the three methods: ANN, PSSM and SVM.

^b^Proteins that have shown affinity to 14-3-3 in high-throughput experiments.

^c^Protein members of 2R-ohnologue families.

Two of the predicted sites, ranked at 2nd and 11th with consensus scores of 1.88 and 1.67, are for family with sequence similarity 122A and 122B (FAM122A and FAM122B). FAM122 is a family of three uncharacterized proteins (A, B and C). FAM122A and FAM122B is a pair of 2R-ohnologues, whereas FAM122C evolved by tandem duplication of FAM122B in mammals (adjacent genes at Xq26.3). Such tandem duplication of 2R-ohnologues is rare (Makino and McLysaght, 2010).

**Fig. 2.**
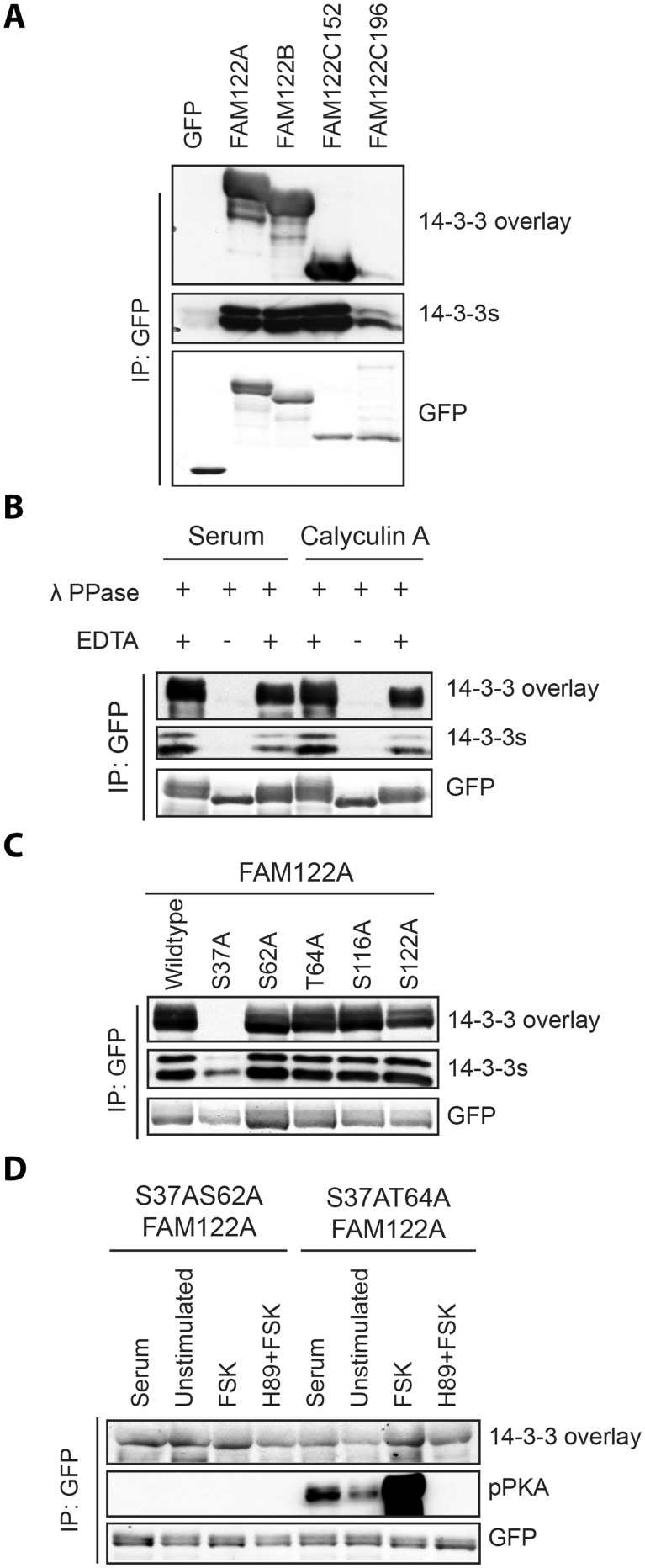
Regulated binding of 14-3-3 proteins to FAM122-GFP proteins. (**A**) HEK293 cells growing in media containing 10% (v/v) serum were transfected to express FAM122A-GFP, FAM122B-GFP and FAM122C-GFP proteins (the latter as 152 and 196 residue isoforms, respectively, excluding the GFP). GFP-tagged proteins isolated from cell lysates (120 mg) with GFP-Trap® were tested for their ability to bind directly to 14-3-3s in Far-Western assays (overlay) and by coimmunoprecipitation of endogenous 14-3-3s (14-3-3s) using the K19 pan-14-3-3 antibody. Anti-GFP signals show levels of the tagged proteins in the immunoprecipitates. (**B**) FAM122A-GFP bound to GFPTrap® was dephosphorylated with lambda phosphatase or not when the phosphatase was inhibited with EDTA. The immunoprecipitates were washed and FAM122A-GFP analyzed for its ability to bind directly to 14-3-3s (overlay) and for retention of co-purified endogenous 14-3-3 proteins (14-3-3s). Cells had been grown in standard medium (serum), with 100 nM calyculin A (a protein phosphatase inhibitor) added for approximately 5 min before lysis, as indicated. (**C**) Wild-type FAM122A-GFP and the indicated serine/threonine-to-alanine mutant proteins were isolated from transfected cells and tested for direct binding to 14-3-3s and for co-immunoprecipitating endogenous 14-3-3s. (**D**) HEK293 cells were transfected to express Ser37Ala/Ser62Ala- FAM122A-GFP and Ser37Ala/Thr64Ala-FAM122A-GFP, as indicated. Cells were serum starved for 10 h, then stimulated with serum (10% (v/v) for 30 min) or forskolin (20 µM for 30 min) with or without H89 pre-treatment (30 µM for 30 min), as indicated. Proteins immunoprecipitated from lysates were tested for 14-3-3 binding (overlay) and coimmunoprecipitation of endogenous 14-3-3 (14-3-3)

Consistent with the 14-3-3-Pred results (Table 2), all three FAM122 family members displayed phosphorylation-dependent binding to 14-3-3 proteins when isolated from transfected cells (Fig. 2A). The binding of 14-3-3 to FAM122A was abolished by its dephosphorylation (Fig. 2B) and by substitution of Ser37 of FAM122A with alanine (Fig. 2C). Although phosphoSer62 and phosphoThr64 of FAM122A also had relatively high 14-3-3-Pred scores (0.614 and 1.076, respectively), mutation of these residues did not affect 14-3-3 binding to FAM122A isolated from cells cultured in standard serum-containing medium (Fig. 2C). However, in the absence of Ser37, stimulating cells with the adenylate cyclase activator forskolin caused a marked increase in 14-3-3 binding to FAM122A, which was abolished when Ser62 was also mutated to alanine and when cells were pre-treated with H89, which is a non-specific cAMP-dependent protein kinase (PKA) inhibitor (Fig. 2D). These data indicate that a 14-3-3 dimer binds to both phosphoSer37 and phosphoSer62 on FAM122A, the latter likely phosphorylated by PKA. Similar experiments showed that 14-3-3 binds to phosphoSer25 and forskolin-regulated phosphoSer50 of FAM122B and to phosphoSer29 (ILRRVN**S**APLI) of FAM122C. Thus, this is an example of a 2R-ohnologue family for which protein members share a conserved 14-3-3-binding ‘lynchpin’. In fact, half of the top 20 candidate proteins (10/20) belong to 2R-ohnologue families.

The benchmark results for the *BLIND* dataset, as well as prediction of 14-3-3-binding sites in the human proteome and the analysis of top high-scoring predictions, suggests the new classifiers developed in this study will be generally useful for identifying potential 14-3-3 sites. Although the methods developed here were not specifically developed to predict pairs of 14-3-3-binding sites due the limited set of proteins for which two binding sites are known, the example of the FAM122 2R-ohnologue family illustrates its use to investigate both primary and secondary 14-3-3-binding phosphosites.

A standalone web server providing a simple yet useful interface to the new methods to score potential Ser/Thr centred motifs for likelihood of binding 14-3-3 proteins is freely available at http://www.compbio.dundee.ac.uk/1433pred. The predictions described here were also integrated in the ANIA database. ANIA adds a functional layer to the peptide-based predictions, by looking for pairs of sites >15 residues apart and by the analysis of sequence alignments of 2R-ohnologue families to identify potential lynchpins (Tinti *et al.*, 2012, 2014). In addition to the human proteome, predictions on proteomes of model organisms, such as *Arabidopsis thaliana*, where several 14-3-3-binding targets have been identified (de Boer *et al*., 2013; Ferl, 1996), will be performed and added to ANIA in the future.

## Supplementary Material

Supplementary Data
