# Supplementary material for: 14-3-3-Pred: improved methods to predict 14-3-3-binding phosphopeptides
Source: Bioinformatics. 2015 Mar 3;31(14):2276–83. doi: 10.1093/bioinformatics/btv133 (PMC4495292; doi:10.1093/bioinformatics/btv133)
Supplement: Supplementary Data [file supp_31_14_2276__index.html]

14-3-3-Pred: improved methods to predict 14-3-3-binding phosphopeptides — 14-3-3-Pred: improved methods to predict 14-3-3-binding phosphopeptides — Supplementary Data 

# 14-3-3-Pred: improved methods to predict 14-3-3-binding phosphopeptides

## Supplementary Data

files

**Files in this Data Supplement:**

- Supplementary Data - xlsx file
